# The E3 ubiquitin ligase TRIM62 and inflammation-induced skeletal muscle atrophy

**DOI:** 10.1186/s13054-014-0545-6

**Published:** 2014-09-29

**Authors:** Franziska Schmidt, Melanie Kny, Xiaoxi Zhu, Tobias Wollersheim, Kathleen Persicke, Claudia Langhans, Doerte Lodka, Christian Kleber, Steffen Weber-Carstens, Jens Fielitz

**Affiliations:** Experimental and Clinical Research Center (ECRC), a Cooperation between Max-Delbrück-Centrum and Charité-Universitätsmedizin Berlin, Campus Buch, Lindenberger Weg 80, 13125 Berlin, Germany; Charité-Universitätsmedizin Berlin, Campus Virchow and Campus Mitte, Anesthesiology and Operative Intensive Care Medicine, Augustenburger Platz 1, 13353 Berlin, Germany; Charité-Universitätsmedizin Berlin, Campus Virchow, Center for Musculoskeletal Surgery, Augustenburger Platz 1, 13353 Berlin, Germany; Cardiology, Charité-Universitätsmedizin Berlin, Campus Virchow, Augustenburger Platz 1, 13353 Berlin, Germany

## Abstract

**Introduction:**

ICU-acquired weakness (ICUAW) complicates the disease course of critically ill patients. Inflammation and acute-phase response occur directly within myocytes and contribute to ICUAW. We observed that tripartite motif–containing 62 (TRIM62), an E3 ubiquitin ligase and modifier of inflammation, is increased in the skeletal muscle of ICUAW patients. We investigated the regulation and function of muscular TRIM62 in critical illness.

**Methods:**

Twenty-six critically ill patients with Sequential Organ Failure Assessment scores ≥8 underwent two skeletal muscle biopsies from the vastus lateralis at median days 5 and 15 in the ICU. Four patients undergoing elective orthopedic surgery served as controls. TRIM62 expression and protein content were analyzed in these biopsies. The kinetics of *Trim62*, *Atrogin1* and *MuRF1* expression were determined in the gastrocnemius/plantaris and tibialis anterior muscles from mouse models of inflammation-, denervation- and starvation-induced muscle atrophy to differentiate between these contributors to ICUAW. Cultured myocytes were used for mechanistic analyses.

**Results:**

TRIM62 expression and protein content were increased early and remained elevated in muscles from critically ill patients. In all three animal models, muscular *Trim62* expression was early and continuously increased. Trim62 was expressed in myocytes, and its overexpression activated the atrophy-inducing activator protein 1 signal transduction pathway. Knockdown of Trim62 by small interfering RNA inhibited lipopolysaccharide-induced interleukin 6 expression.

**Conclusions:**

TRIM62 is activated in the muscles of critically ill patients. It could play a role in the pathogenesis of ICUAW by activating and maintaining inflammation in myocytes.

**Trial registration:**

Current Controlled Trials ID: ISRCTN77569430 (registered 13 February 2008)

**Electronic supplementary material:**

The online version of this article (doi:10.1186/s13054-014-0545-6) contains supplementary material, which is available to authorized users.

## Introduction

ICU-acquired weakness (ICUAW) is a devastating complication of critical illness characterized by loss of muscle mass [1], preferential atrophy of fast-twitch myofibers and weakness [2-4]. Affected patients face a prolonged hospital stay and mechanical ventilation, increased hospital mortality and chronic physical disability [5,6]. The pathophysiology of ICUAW is poorly understood [7]. However, we [8] and others [1] have shown that dysbalanced muscular protein homeostasis due to increased protein degradation and reduced protein synthesis occurs in muscle of critically ill patients and may contribute to ICUAW [1,2,8,9]. Breakdown of muscular proteins such as myosin heavy chain (MyHC) is mediated by the ubiquitin-proteasome system (UPS) [10], which is activated in muscle of critically ill patients [1,8,11] and involves the F-box adaptor protein FBXO32/Atrogin1 [12] and the E3 ubiquitin ligase muscle RING (really interesting new gene) finger–containing protein 1 (MuRF1). Atrogin1 and MuRF1 are rapidly and transiently increased in the skeletal muscle of critically ill patients [8]. However, muscle atrophy and regulation of *Atrogin1* and *MuRF1* expression are not synchronized, because atrophy occurs later in the disease process, when *Atrogin1* and *MuRF1* have already returned to baseline [8]. This discrepancy argues for additional continuously activated atrophy pathways. Chronic and persistent inflammation and acute-phase response directly occurring in the skeletal muscle of critically ill patients might be one of these mechanisms [13]. Recently, we have shown that interleukin 6 (IL-6) and the acute-phase response proteins serum amyloid A1 (SAA1) and SAA4 are continuously elevated in the muscle of critically ill patients [13]. Both IL-6 [14,15] and SAA1 [16,17] are known to induce atrophy by increasing protein degradation in the skeletal muscle of both patients and rodents. We performed a gene expression array and found the modifier of inflammation tripartite motif–containing 62 (TRIM62) to be increased in the muscle of critically ill patients [13]. TRIM62 belongs to the family of RING finger E3 ubiquitin ligases [18,19] and was identified as a dominant regulator of acinar morphogenesis in the mammary gland [20]. Strong evidence exists that TRIM62 plays a role in Toll-like receptor 4 (TLR4) signaling. More specifically, TRIM62 activates the Toll/interleukin 1 receptor domain–containing adapter inducing interferon β (TRIF) branch of the TLR4 signaling pathway, leading to increased activity of the activator protein 1 (AP-1) transcription factor in primary macrophages [21]. Because AP-1 signaling is essential for denervation-induced atrophy [22], we hypothesized that TRIM62-mediated activation of AP-1 signaling in myocytes contributes to inflammation-induced atrophy in critically ill patients. To specifically focus on early time points of muscle atrophy and to differentiate between the major contributors of ICUAW, we relied on three mouse atrophy models described elsewhere: cecal ligation and puncture (CLP) mimicking sepsis, denervation-induced atrophy and food deprivation [13]. These models were used to compare the kinetics of *Trim62* with *Atrogin1* and *MuRF1* gene expression in muscle. Cultured myocytes and reporter gene assays were used for mechanistic analyses.

## Material and methods

### Patients

The institutional review board of the Charité approved the study, and written informed consent was obtained from the patient or the patient’s legal proxy (Charité EA2/061/06). We recently reported clinical data and molecular analyses in the biopsy specimens of the same patients [2,8,13,23]. We specifically included patients at high risk of developing ICU-acquired muscle wasting and weakness [24]. Open muscle biopsies from the vastus lateralis were performed at median day 5 in 26 ICU patients (early time point). Of these 26 patients, 14 remained at least to median day 15 in the ICU (late time point), when a second biopsy specimen from the vastus lateralis was obtained. Four age- and gender-matched patients undergoing elective orthopedic surgery, otherwise healthy, permitted a biopsy from the vastus lateralis at the time point of elective surgery. For further details, refer to Additional file 1.

### Animal models of muscle atrophy

To focus on early time points of muscle atrophy, we relied on three mouse models described elsewhere: CLP surgery that mimics sepsis, denervation-induced atrophy and food deprivation [13,25,26]. All animal procedures were performed in accordance with the guidelines of the Max-Delbrück Center for Molecular Medicine and the Charité-Universitätsmedizin Berlin and were approved by the Landesamt für Gesundheit und Soziales (LaGeSo, Berlin, Germany) for the use of laboratory animals (permit number G 0129/12). They followed the principles of laboratory animal care set forth by the National Institutes of Health (NIH) in the *Guide for the Care and Use of Laboratory Animals* (NIH Publication 86-23, revised 1985), as well as the current version of the German Law on the Protection of Animals. Briefly, 6- to 8-week-old male C57BL/6 N mice were used for all experiments. CLP surgery was performed to induce polymicrobial sepsis according to a published protocol [25] and as recently reported [13]. Sham mice were treated identically, except for the ligation and puncture of the cecum. CLP (*n* =4 or 5) and sham-treated (*n* =4 or 5) mice were sacrificed 24 hours, 48 hours, 72 hours or 96 hours after surgery. Neurogenic atrophy was induced by dissection of the left sciatic nerve (denervation). The sciatic nerve of the right leg was cut, and a 3-mm piece was excised (denervated). The right leg remained innervated and was used as the control (innervated). Mice were sacrificed at baseline or 7 days, 14 days or 21 days postsurgery (*n* =6 each). Food deprivation was performed for 0 hours (control), 24 hours or 48 hours (*n* =6 each).

For detailed information about animal experiments, quantitative RT-PCR (qRT-PCR), immunohistology, immunoblotting, myoblast culture and immunocytology, small interfering RNA (siRNA) transfection and luciferase reporter assay, please refer to Additional files 1.

### Statistical tests

A two-sided Mann-Whitney *U* test was used to determine statistical differences. Data shown are mean ± SEM. Statistical tests were calculated using GraphPad Prism software (GraphPad Software, La Jolla, CA, USA). Box plots showing medians with 25th and 75th percentiles were made using GraphPad Prism 5. *P* <0.05 was considered statistically significant.

## Results

### TRIM62 mRNA was upregulated in muscle of critically ill patients

**Figure 1:**
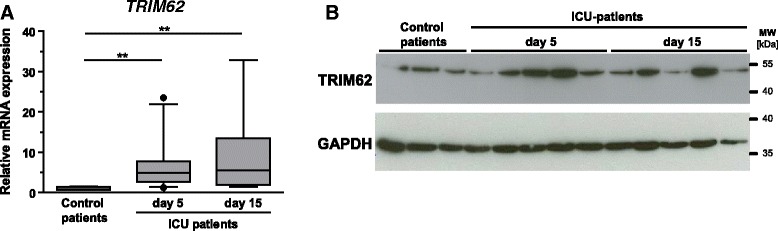
**Muscular** ***TRIM62*** **expression in critically ill patients. (A)** Quantitative RT-PCR analyses of tripartite motif–containing 62 (*TRIM62*) expression in vastus lateralis muscles of orthopedic control patients (*n* =4) and patients with ICU-acquired weakness (ICUAW) at early (day 5, *n* =26) and late (day 15, *n* =14) time points. Glyceraldehyde 3-phosphate dehydrogenase (*GAPDH*) expression was used as the reference. Data shown are relative expression and fold changes compared to control patients. Data presented in box plots are medians and 25th and 75th percentiles. **P <0.01. **(B)** Immunoblots of proteins from vastus lateralis muscles of orthopedic control and critically ill patients at early (day 5) and late (day 15) time points using anti-TRIM62 antibody. GAPDH was used as loading control.

The study protocol (Additional file 2: Figure S1), data on patients’ characteristics (Additional file 1: Table S1) and treatment (Additional file 1: Table S2) are provided. The data from our microarray have been deposited in the National Center for Biotechnology Information Gene Expression Omnibus (GEO) database [GEO:GSE53702] (http://www.ncbi.nlm.nih.gov/geo/query/acc.cgi?acc=GSE53702). We found a 3.9-fold upregulation of *TRIM62* [13]. We performed qRT-PCR in a subset of our recently published ICU patients [8] at the early (day 5, *n* =26) and late (day 15, *n* =14) time points and orthopedic controls (*n* =4) to confirm these data. Continuous upregulation of muscular *TRIM62* mRNA was confirmed for both time points of critical illness (*P* <0.01) (Figure 1A). Immunoblot analysis showed that muscular TRIM62 protein was increased at the early time point and remained elevated until the late time point in critically ill patients (Figure 1B). In contrast, *Atrogin1* and *MuRF1* expression was significantly increased in the early and returned to control levels in the late biopsy specimens in these patients as recently reported [8].

### Trim62 was persistently elevated during skeletal muscle atrophy in mice

Because Trim62 has been shown to be involved in inflammatory response of immune cells [21], we reasoned that Trim62 could play a role in the development of ICUAW. We used three standard animal models of muscle atrophy—CLP [13,25], denervation [26] and food deprivation—to investigate regulation of *Trim62* and to compare it with the expression of the standard atrophy markers *Atrogin1* and *MuRF1*.

### Muscular *Trim62* expression was increased during inflammation-induced skeletal muscle atrophy in mice

**Figure 2:**
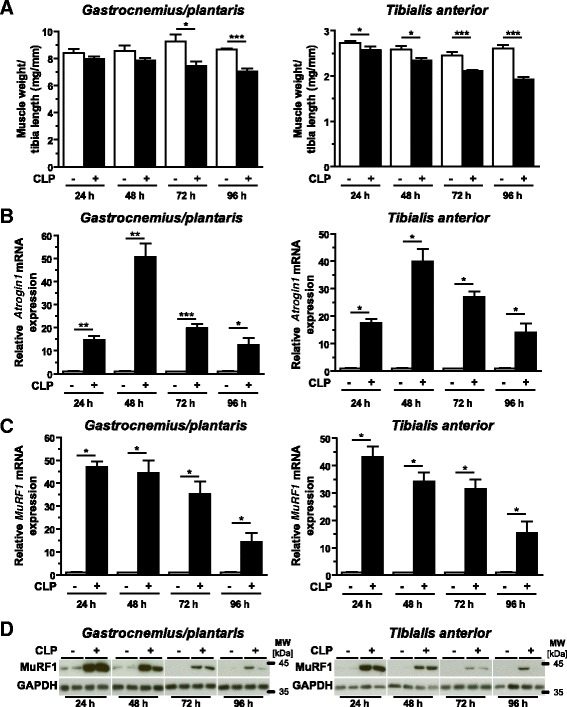
**Inflammation leads to skeletal muscle atrophy** ***in vivo*** **.** Six- to eight-week-old male C57BL/6 N mice were subjected to sham operations (sham, *n* =5) or cecal ligation and puncture (CLP) surgery (*n* =5), as indicated, to induce polymicrobial sepsis. **(A)** Weights of the gastrocnemius/plantaris and tibialis anterior muscles were determined after 24 hours, 48 hours, 72 hours or 96 hours and normalized to tibia length. Data are presented as mean ± SEM. ***P <0.001, *P <0.05. Quantitative RT-PCR analyses of *Atrogin1* **(B)** and *MuRF1* **(C)** expression, and immunoblotting of proteins using anti-MuRF1 antibody **(D)**, in gastrocnemius/plantaris and tibialis anterior muscles at 24 hours, 48 hours, 72 hours or 96 hours after surgery, as indicated. Glyceraldehyde 3-phosphate dehydrogenase (Gapdh) expression and protein content were used as reference values, and data shown are relative changes. Data are presented as mean ± SEM. ***P <0.001, **P <0.01, *P <0.05. MuRF1, Muscle RING (really interesting new gene) finger–containing protein 1.

Inflammation-induced muscle atrophy was induced by the CLP method of polymicrobial sepsis as previously described [13,25]. Recently, we reported that increased expression of *Il-6* and *Saa1* in muscles of CLP mice might contribute to inflammation-induced atrophy [13,25]. In the present study, general inflammation was confirmed by an early and persistent increase in gene expression of the inflammatory cytokines *Il-6* and tumor necrosis factor α (*Tnf-α*), as well as of the acute-phase protein Saa1, in the liver of CLP mice (Additional file 3: Figure S2). Septic mice showed a significant decrease in body weight as soon as 24 hours after surgery. CLP mice continued their weight loss, which reached its maximum after 96 hours at a reduction of 19% of body weight (*P* <0.05) (Additional file 4: Figure S3A). A significant decrease in muscle weights was found after 72 hours and 96 hours of sepsis, indicative of muscular atrophy (Figure 2A). Using qRT-PCR, we found a significant increase in *Atrogin1* and *MuRF1* expression after 24 hours of sepsis in gastrocnemius/plantaris and tibialis anterior muscles. *Atrogin1* and *MuRF1* expression followed a time course with early induction and a subsequent decrease (Figures 2B and 2C). Whereas *MuRF1* expression was highest at the 24-hour time point, the induction of *Atrogin1* peaked at 48 hours of sepsis (Figures 2B and 2C). Immunoblot analysis confirmed that muscular MuRF1 protein content went in parallel with its expression, showing an early induction and a subsequent decrease in gastrocnemius/plantaris and tibialis anterior muscles of CLP mice (Figure 2D).

**Figure 3:**
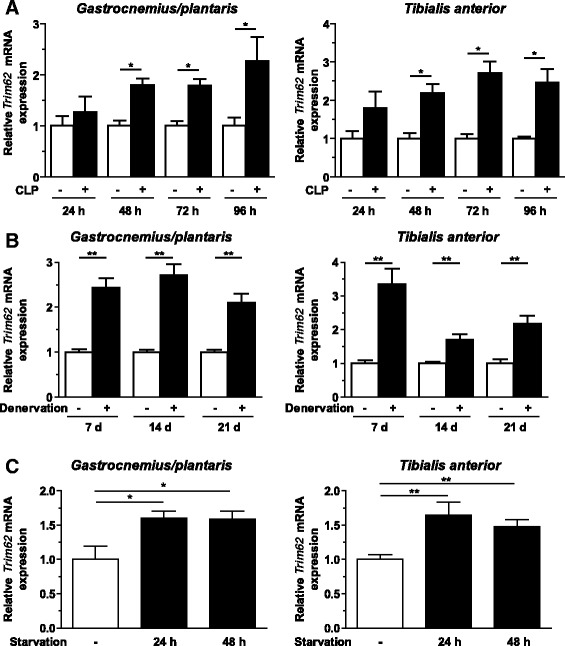
***Trim62*** **expression is increased during inflammation-, denervation- and starvation-induced muscle atrophy. (A)** Quantitative RT-PCR (qRT-PCR) analyses of *Trim62* expression in gastrocnemius/plantaris and tibialis anterior muscles at 24 hours, 48 hours, 72 hours and 96 hours after surgery, as indicated. CLP, Cecal ligation and puncture surgery. **(B)** qRT-PCR analyses of *Trim62* expression in gastrocnemius/plantaris and tibialis anterior muscles 7 days, 14 days and 21 days after denervation or sham surgery. **(C)** qRT-PCR analyses of *Trim62* expression in gastrocnemius/plantaris and tibialis anterior muscles of control mice (–, *n* =6) and mice deprived of food for 24 hours (*n* =6) or 48 hours (*n* =6), as indicated. Glyceraldehyde 3-phosphate dehydrogenase (*Gapdh*) expression was used as the reference in all assays. Data shown are fold changes of expression in sham-operated and untreated mice. Data are presented as mean ± SEM. **P <0.01, *P <0.05.

Muscular *Trim62* expression was measured by qRT-PCR. It was significantly increased in gastrocnemius/plantaris and tibialis anterior muscles at 48 hours and 72 hours after CLP surgery. Throughout the experiment, *Trim62* expression levels were persistently elevated in both muscle types (Figure 3A).

### Muscular Trim62 expression was increased in denervation-induced skeletal muscle atrophy in mice

**Figure 4:**
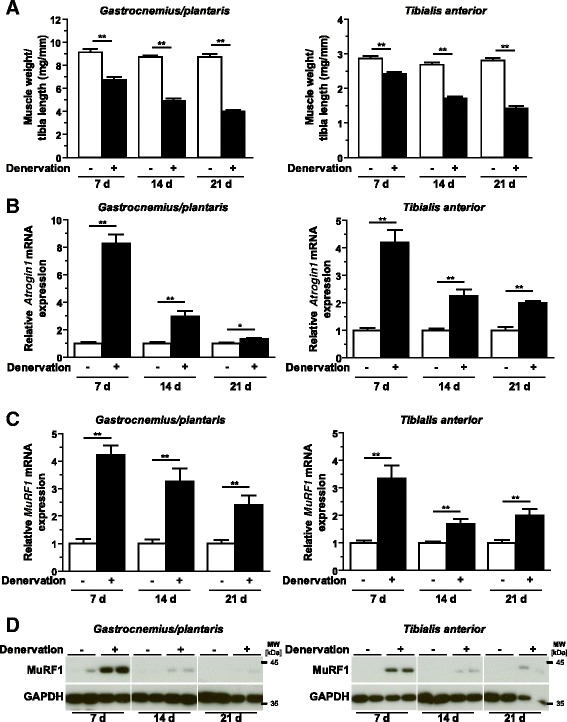
**Denervation-induced atrophy.** Six- to eight-week-old male C57BL/6 N mice were subjected to surgery. The sciatic nerve of the left hindlimb was dissected (denervated, *n* =6), and a sham procedure was performed at the right side (innervated, *n* =6), as indicated. **(A)** The weights of gastrocnemius/plantaris and tibialis anterior muscles normalized to tibia length were determined at baseline (–) and after 7 days, 14 days and 21 days. Data are presented as mean ± SEM. **P <0.05. Quantitative RT-PCR analyses of *Atrogin1* **(B)** and *MuRF1* **(C)** expression and immunoblotting of proteins using anti-MuRF1 antibody **(D)** in gastrocnemius/plantaris and tibialis anterior muscles at 7 days, 14 days and 21 days after surgery, as indicated. Glyceraldehyde 3-phosphate dehydrogenase (Gapdh) expression and protein content were used as reference values, and data shown are the fold changes of the respective innervated sites. Data are presented as mean ± SEM. **P <0.01, *P <0.05. MuRF1, Muscle RING (really interesting new gene) finger–containing protein 1.

We investigated if *Trim62* was regulated in neurogenic muscle atrophy. A progressive loss of muscle weight was observed for gastrocnemius/plantaris and tibialis anterior muscles. Weights of gastrocnemius/plantaris and tibialis anterior muscles were significantly decreased after 7 days of denervation (Figure 4A). A continuous decrease in muscle mass was observed until 21 days of denervation, reaching 54% and 49% (*P* <0.01 for both) of muscle weight for gastrocnemius/plantaris and tibialis anterior muscles, respectively (Figure 4A). *Atrogin1* (Figure 4B) and *MuRF1* (Figure 4C) expression was significantly increased at both time points in gastrocnemius/plantaris and tibialis anterior muscles*.* More specifically, *Atrogin1* and *MuRF1* expression was highest after 7 days of denervation. *Atrogin1* expression was 8.3- and 4.2-fold upregulated in gastrocnemius/plantaris and tibialis anterior muscles, respectively, after 7 days of denervation (*P* <0.01 for both) (Figure 4B). At the same time point, *MuRF1* expression was increased four- and threefold in gastrocnemius/plantaris and tibialis anterior muscles, respectively (*P* <0.01 for both) (Figure 4C). The MuRF1 protein content showed a comparable time course during denervation in gastrocnemius/plantaris and tibialis anterior muscles (Figure 4D).

*Trim62* expression was also significantly increased at both time points in denervated gastrocnemius/plantaris and tibialis anterior muscles (Figure 3B). In gastrocnemius/plantaris muscles, a 2.4-fold upregulation of *Trim62* was measured 7 days after denervation, and it remained at this level at 14 days and 21 days (*P* <0.01 for all). In tibialis anterior muscle, *Trim62* expression reached its maximum at 7 days (3.3-fold induction) and decreased thereafter (1.7-fold after 14 days and 2.2-fold after 21 days) (*P* <0.01 for both) (Figure 3B).

### Starvation-induced atrophy was accompanied by increased *Trim62* expression in mouse skeletal muscle

**Figure 5:**
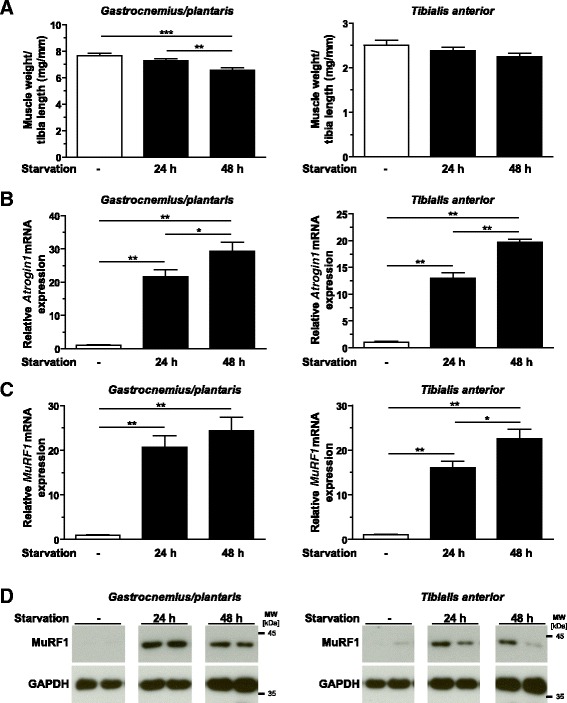
**Starvation leads to skeletal muscle atrophy** ***in vivo*** **.** Six- to eight-week-old male C57BL/6 N mice were subjected to food deprivation for 24 hours (*n* =6) or 48 hours (*n* =6), as indicated. Mice fed standard chow (controls, –; *n* =6) were used as controls. **(A)** Weights of gastrocnemius/plantaris and tibialis anterior muscles normalized to tibia length are shown. Data are presented as mean ± SEM. ***P <0.001, **P <0.01. Quantitative RT-PCR analyses of *Atrogin1* **(B)** and *MuRF1* **(C)** expression, and immunoblotting of proteins using anti-MuRF1 antibody **(D)**, in gastrocnemius/plantaris and tibialis anterior muscles of control animals (–) and mice deprived of food for 24 hours and 48 hours are shown, as indicated. Glyceraldehyde 3-phosphate dehydrogenase (Gapdh) expression and protein content were used as reference values, and data shown are fold changes compared to controls. Data are presented as mean ± SEM. **P <0.01, *P <0.05. MuRF1, Muscle RING (really interesting new gene) finger–containing protein 1.

To investigate the impact of starvation on muscle atrophy, mice were food-deprived for 24 hours and 48 hours. Food deprivation led to decreases in body weight (14% after 24 hours (*P* <0.01) and 20% after 48 hours (*P* <0.01)) (Additional file 4: Figure S3B). Following food deprivation, a reduction in muscle weight was found for the gastrocnemius/plantaris muscles (15% after 48 hours (*P* <0.001) of starvation), but not tibialis anterior muscle (Figure 5A). qRT-PCR experiments showed an upregulation of muscular *Atrogin1* and *MuRF1* expression during starvation (Figures 5B and 5C). *Atrogin1* expression increased 21.6-fold and 29.4-fold after 24 hours and 48 hours of starvation, respectively, in gastrocnemius/plantaris muscles (*P* <0.01 for both). *Atrogin1* expression was also increased in tibialis anterior muscle (Figure 5B). In gastrocnemius/plantaris muscles, *MuRF1* expression increased 21-fold and 24-fold after 24 hours and 48 hours of starvation, respectively (*P* <0.01 for both). *MuRF1* expression was also increased in tibialis anterior muscle (Figure 5C). The MuRF1 protein content was increased in gastrocnemius/plantaris and tibialis anterior muscles of mice subjected to starvation (Figure 5D).

Fasting resulted in a significant increase in muscular *Trim62* expression after 24 hours and 48 hours of starvation (Figure 3C). *Trim62* expression increased 1.6-fold after 24 hours (*P* <0.05) and remained at this level at 48 hours (*P* <0.05) of starvation in gastrocnemius/plantaris and tibialis anterior muscles (*P* <0.01 for both).

### Trim62 contributes to inflammatory response in muscle cells

**Figure 6:**
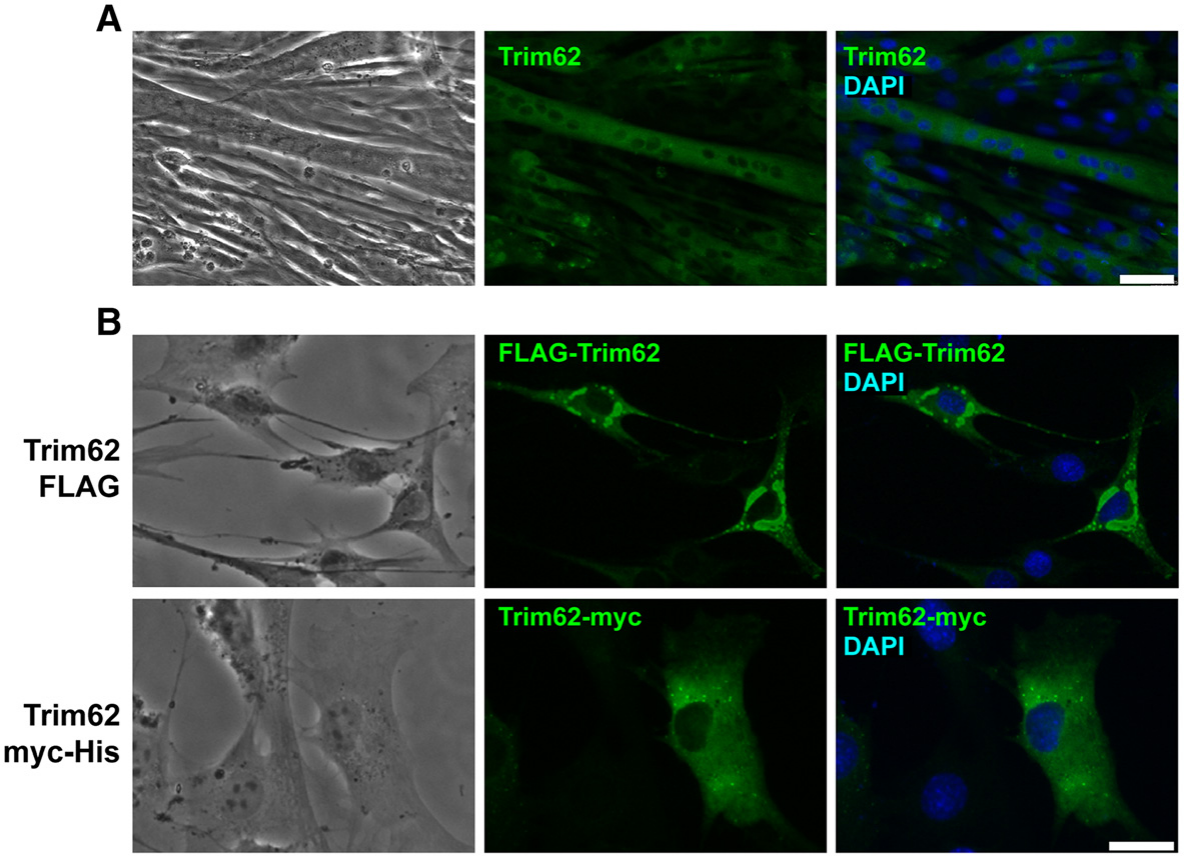
**Trim62 is expressed in myocytes. (A)** Immunocytochemistry of differentiated C2C12 myotubes was performed using an anti-Trim62 antibody. Nuclei were stained with 4′,6-diamidino-2-phenylindole (DAPI, blue). Scale bar, 50 μm. **(B)** C2C12 myoblasts were transfected with expression plasmids encoding Trim62-FLAG (top panel), Trim62 myc/His-6 (bottom panel) or an empty vector control plasmid. Immunofluorescence analysis was performed using anti-FLAG and anti-Myc antibodies, respectively, as well as an Alexa Fluor 488–coupled secondary antibody. Nuclei were stained with DAPI (blue). Scale bar, 25 μm.

Immunocytochemistry was performed to elucidate whether endogenous Trim62 protein was contained in myocytes. As expected, differentiated C2C12 myotubes contained Trim62 endogenously. Trim62 was exclusively localized in the cytoplasm of these cells (Figure 6A). The localization of Trim62 in the cytoplasm was confirmed when we transfected expression plasmids encoding Trim62 with either an amino-terminal FLAG tag or a carboxy-terminal myc/His-6 tag in C2C12 myoblasts (Figure 6B). These data indicate that Trim62 is contained in myocytes, where it is localized mainly in the cytoplasm.

**Figure 7:**
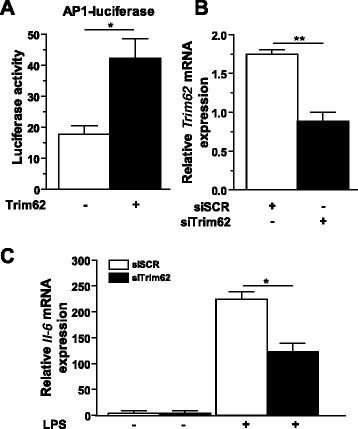
**Trim62 is involved in atrophy signaling. (A)** Luciferase assays were performed on cell extracts of HEK293 cells transfected with the activator protein 1 (AP-1) luciferase reporter plasmid, along with expression plasmids encoding the Trim62 protein (+) or empty (–) expression plasmid. Values were normalized to expression of CMV-LacZ and are expressed as the relative luciferase activity to CMV-LacZ ratio with the Trim62 expression plasmid compared to the reporter alone. Data are shown as mean ± SEM. *P <0.05. **(B)** Differentiated C2C12 myotubes were transfected with control scrambled small interfering RNA (siSCR) or siRNA against Trim62 (siTrim62) (25 nM each), as indicated. Cells were harvested 24 hours after transfection, and *Trim62* expression was quantified using quantitative RT-PCR analysis. Glyceraldehyde-3 phosphate dehydrogenase (*Gapdh*) expression was used as the reference value. Data are presented as mean ± SEM. **P <0.01. **(C)** Differentiated C2C12 myotubes were transfected with siSCR or siTrim62 (25 nM each) and treated with vehicle (–) or lipopolysaccharide (LPS, +, 100 μM, for 2 hours), as indicated. RT-PCR was used to quantitate *Trim62* expression. *Gapdh* expression was used as the reference value. Data are presented as mean ± SEM. *P <0.05. IL-6, Interleukin 6.

Trim62 was implicated in the inflammatory response of immune cells by regulating the TLR4 signaling pathway, leading to activation of AP-1 [21]. Therefore, we analyzed whether overexpressed Trim62 affects a reporter construct harboring three consecutive AP-1 consensus sites. Indeed, overexpression of Trim62 induced the AP-1-dependent promoter construct, indicating that Trim62 activates AP-1-dependent signaling events (Figure 7A). We also tested whether deletion of Trim62 in differentiated C2C12 myotubes by siRNA has an effect on TLR4-mediated activation of AP-1. Following knockdown of Trim62 in C2C12 myotubes (Figure 7B), we treated these cells with the TLR4 agonist lipopolysaccharide (LPS) and analyzed *Il-6* expression by qRT-PCR. As expected, LPS treatment led to increased *Il-6* expression in myocytes (Figure 7C). When Trim62 was downregulated, the LPS-mediated increase in *Il-6* expression was diminished. These data indicate that Trim62 is involved in LPS-induced *Il-6* expression and thus in the inflammatory response in myocytes.

## Discussion

Increased *TRIM62* gene expression and protein content were found in skeletal muscle of critically ill patients. This finding was confirmed by increased *Trim62* expression in skeletal muscle of three muscle atrophy mouse models. In contrast to decreasing levels of *Atrogin1* and *MuRF1* during the late phase of muscle atrophy, *Trim62* levels remained continuously elevated in all atrophy models. These data implicate a role of Trim62 for the later phase of muscle atrophy. Because Trim62 activates the TRIF branch of TLR4 signaling which leads to increased LPS-induced *IL-6* expression [21], we propose that continuous Trim62 activation contributes to persistent inflammation in muscle, promoting atrophy and development of ICUAW in critically ill patients. To our knowledge, the present study is the first in which TRIM62 regulation has been investigated in human and mouse skeletal muscle during atrophy.

TRIM62 belongs to the TRIM family of RING finger E3 ubiquitin ligases [18,19] that is involved in the regulation of differentiation, immunity, development and apoptosis [18]. This protein family is also involved in muscular protein homeostasis. In muscle, TRIM mutations lead to primary myopathies. For instance, mutated *TRIM32* leads to limb-girdle muscular dystrophy type 2H [27]; mutations in *TRIM18* (MID1) cause Opitz G/BBB syndrome [28]; and *TRIM63*/MuRF1 mutations result in hypertrophic cardiomyopathy [29]. The MuRF family (MuRF1/*TRIM63*, MuRF2/*TRIM55* and MuRF3/*TRIM54*) of TRIM proteins has essential functions for protein homeostasis in striated muscle responsible for myogenesis [30] and maintenance [10,31-33]. Our data indicate that TRIM62 is also involved in muscular protein homeostasis, especially during inflammation-induced atrophy. We have established TRIM62 as novel atrophy marker. However, further studies are needed to understand its importance in muscle atrophy.

Critically ill patients often develop ICUAW as a severe complication of critical illness. It affects more than half of all ICU patients [34]. ICUAW is characterized by skeletal muscle atrophy and weakness [2-4], is associated with elevated morbidity and mortality and impairs short- and long-term clinical outcomes [6,35]. Recently, we found persistently increased *IL-6* expression and elevated SAA1 content in muscles of critically ill patients [13,24]. On the basis of these data, we hypothesized that continuous inflammation and acute-phase response in muscle play an important role in ICUAW [13,24]. Our observation that TRIM62 is persistently increased in muscles of critically ill patients at high risk of developing ICUAW and in all atrophy mouse models strengthens this hypothesis. Knockdown of TRIM62 in primary macrophages abolished TRIF-mediated AP-1 signal transduction following LPS treatment [21]. We show that this pathway is also active in myocytes in our finding that knockdown of Trim62 inhibited LPS-induced *Il-6* expression in C2C12 cells. These proinflammatory actions of TRIM62 might explain our recent finding of persistently increased *IL-6* expression in muscles of ICUAW patients [13,24]. Because AP-1 signaling is essential in denervation-induced atrophy [22], TRIM62-mediated AP-1 activation is particularly important. We confirm that Trim62 is involved in this cascade, because its overexpression increased AP-1 activity in myocytes. We propose that persistent activation of TRIM62 in muscle could lead to chronically increased AP-1 activity, promoting atrophy even when Atrogin1 and MuRF1 expression have normalized.

An imbalanced muscular protein homeostasis with increased UPS-mediated protein degradation and reduced protein synthesis plays a dominant role in critically ill patients [1,8]. Atrogin1 and MuRF1 are key factors in this pathway and are consistently used as atrophy markers [12]. Atrogin1 and MuRF1 are rapidly, but only transiently, increased in muscles of critically ill patients [8] and in atrophy mouse models [12]. This time course of *Atrogin1* and *MuRF1* expression is consistent with our findings reported here. In contrast, muscular *Trim62* expression remained increased throughout the disease course in ICUAW patients and in all mouse models. These findings support the assumption that Atrogin1 and MuRF1 are predominantly involved in the early phase of atrophy. We suggest that early and sustained muscular *Trim62* expression continuously activate atrophy signaling pathways involved in later phases of the disease process. Increased muscular *Trim62* expression in all atrophy models implicates that *Trim62* upregulation is a general and nonspecific feature in muscle atrophy.

### Limitations

We used CLP to investigate inflammation-induced muscle atrophy. This method causes polymicrobial sepsis and systemic inflammation in mice. Therefore, we cannot define a precise pathomechanism (that is, specific inflammatory cytokines, bacteria or fungi) responsible for inflammation-induced muscle atrophy. However, CLP is considered a gold standard in sepsis research [25,36,37]. The pathomechanisms and cytokine release following CLP are close to the human situation in sepsis [38-40]. Further studies are needed to investigate specific pathways in inflammation-induced atrophy.

The balance between protein synthesis and degradation responsible for maintenance of muscular protein homeostasis is disturbed in critically ill patients [1]. In our present study, we found an increased atrogene gene expression indicative for enhanced UPS-dependent protein degradation and muscle atrophy [12,41,42]. However, we did not investigate whether protein synthesis was reduced in our models. Therefore, we cannot draw any conclusions about whether muscle atrophy occurred because of increased protein degradation alone or whether an accompanying decrease in protein synthesis contributed to this phenotype.

## Conclusions

Chronic inflammation and acute-phase response in muscle plays an important role in ICUAW pathogenesis [13,24]. TRIM62 might be a new factor responsible for persistent inflammation in muscle that promotes muscle atrophy and ICUAW. *TRIM62* gene expression and protein content were increased in muscles of critically ill patients and three standard murine muscle atrophy models. Continuously increased muscular *TRIM62* expression throughout the disease course in ICUAW patients and in all mouse models was in clear contrast to the time course of the commonly used atrophy markers Atrogin1 and MuRF1, which are rapidly, but only transiently, increased in muscles of critically ill patients [8] and murine muscle atrophy models [12]. Sustained muscular *TRIM62* expression might point toward its role in later phases of muscle atrophy, whereas Atrogin1 and MuRF1 are involved predominantly in the early disease phase. With these data, we establish TRIM62 as a novel marker for muscle atrophy. In summary, we think that Trim62 contributes to inflammation-induced muscle atrophy at least in part by activation of the AP-1 signal transduction pathway.

## Key messages

- Persistent inflammation and acute-phase response in muscle contributes to the pathogenesis of ICUAW.
- The E3 ubiquitin ligase and activator of inflammation TRIM62 is continuously increased in skeletal muscle of ICUAW patients and mouse models of inflammation-induced atrophy.
- In contrast to bona fide atrogenes, TRIM62 expression remains increased in atrophic muscle throughout the disease process.
- Overexpression of Trim62 activated the atrophy-inducing AP-1 signal transduction pathway in myocytes, and its knockdown inhibited LPS-induced *Il-6* expression.
- TRIM62 could play a role in ICUAW pathogenesis by activating and maintaining inflammation in muscle.

## Additional files

Additional file 1: Table S1. Patient characteristics. ALI/ARDS, Acute lung injury/acute respiratory distress syndrome; BMI, Body mass index; CNS, Central nervous system; ICU, Intensive care unit; MRC, Medical Research Council; NA, Not applicable; ND, Not determined; SOFA, Sequential Organ Failure Assessment score; SAPS-II, Simplified Acute Physiology Score II. **Table S2.** Treatment of ICU patients during study period. RASS, Richmond Agitation Sedation Scale; PBW, Predicted body weight. Data shown are median (IQR) or number and percentage. **Table S3.** Primer pairs for quantitative RT-PCR are shown. TRIM62, Tripartite motif–containing 62; Saa1, Serum amyloid A1; MuRF1, Muscle RING (really interesting new gene) finger–containing protein; GAPDH, Glyceraldehyde 3-phosphate dehydrogenase; IL-6, Interleukin 6; Tnfα, Tumor necrosis factor α; Hs, Homo sapiens; Mm, *Mus musculus*. Additional file 2: Figure S1. Study protocol. Additional file 3: Figure S2. CLP-induced inflammation and acute-phase response in liver. Quantitative RT-PCR analyses of interleukin 6 (*IL-6*), tumor necrosis factor α (*Tnfα*) and serum amyloid A1 (*Saa1*) expression in the liver 24 hours, 48 hours, 72 hours or 96 hours after surgery, as indicated. Glyceraldehyde 3-phosphate dehydrogenase expression was used as a reference, and data are shown as relative expression. Data presented are mean ± SEM. ***P* <0.01, **P* <0.05. Additional file 4: Figure S3. Body weight during skeletal muscle atrophy. Body weight normalized to tibia length is shown for skeletal muscle atrophy induced by CLP **(A)**, starvation **(B)** and denervation **(C)**. Data presented are mean ± SEM. ***P* <0.01, **P* <0.05.

## Abbreviations

AP-1: Activator protein 1
CLP: Cecal ligation and puncture
ICUAW: ICU-acquired weakness
Il-6: Interleukin 6
MuRF: Muscle RING (really interesting new gene) finger–containing protein
MyHC: Myosin heavy chain
qRT-PCR: Quantitative real-time polymerase chain reaction
SAA: Serum amyloid A
TLR4: Toll-like receptor 4
TRIF: Toll/interleukin 1 receptor domain–containing adapter inducing interferon β
Trim: Tripartite motif–containing
