# Supplementary material for: The E3 ubiquitin ligase TRIM62 and inflammation-induced skeletal muscle atrophy
Source: Crit Care. 2014 Sep 29;18(5):545. doi: 10.1186/s13054-014-0545-6 (PMC4231194; doi:10.1186/s13054-014-0545-6)
Supplement: Additional file 1: Table S1. — Patient characteristics. ALI/ARDS, Acute lung injury/acute respiratory distress syndrome; BMI, Body mass index; CNS, Central nervous system; ICU, Intensive care unit; MRC, Medical Research Council; NA, Not applicable; ND, Not determined; SOFA, Sequential Organ Failure Assessment score; SAPS-II, Simplified Acute Physiology Score II. Table S2. Treatment of ICU patients during study period. RASS, Richmond Agitation Sedation Scale; PBW, Predicted body weight. Data shown are median (IQR) or number and percentage. Table S3. Primer pairs for quantitative RT-PCR are shown. TRIM62, Tripartite motif–containing 62; Saa1, Serum amyloid A1; MuRF1, Muscle RING (really interesting new gene) finger–containing protein; GAPDH, Glyceraldehyde 3-phosphate dehydrogenase; IL-6, Interleukin 6; Tnfα, Tumor necrosis factor α; Hs, Homo sapiens; Mm, Mus musculus. [file 13054_2014_545_MOESM1_ESM.doc]

**Additional file 1**

**Methods**

**Patients**

The institutional review board (IRB) of the Charité approved the study and written informed consent was obtained from legal proxy or from the study subject (Charité EA2/061/06). We recently reported on molecular analyses in the biopsy specimens of the same patients [1-4]. We specifically included patients at high risk to develop ICU-acquired muscle wasting and weakness [5]. Accordingly, critically ill, mechanically ventilated ICU-patients were eligible for study inclusion once they showed SOFA scores ≥8 on three consecutive days within the first five days after ICU admission. Not included by prior definition were patients <18 years of age, receiving treatment for longer than 7 days, a body mass index >35 kg/m^2^, pre-existing neuromuscular diseases, insulin-dependent diabetes mellitus, and moribund patients. ICU-patients received treatment according to standard procedures including goal-directed sedation treatment and sepsis guidelines. In addition, physiotherapy by an experienced physiotherapist starting from day 1 in ICU was performed, including passive range of motion, positioning, and active exercises as soon as possible. Open muscle biopsy from the *vastus lateralis* was performed at median day 5 from 26 ICU patients (early time point). Of these 26 patients 14 remained at least to median day 15 in ICU (late time point), when a second biopsy specimen from the *vastus lateralis* was obtained. Four age and gender-matched patients undergoing elective orthopedic surgery, elsewise healthy, permitted a biopsy from the *vastus lateralis* at the time point of elective surgery. Trial registration: http://www.controlled-trials.com, ISRCTN77569430.

**Animal models.**

All animal procedures, cecal ligation and puncture surgery, food deprivation and denervation, were performed in accordance with the guidelines of the Max-Delbrück Center for Molecular Medicine and the Charité-Universitätsmedizin Berlin, and were approved by the Landesamt für Gesundheit und Soziales (LaGeSo, Berlin, Germany) for the use of laboratory animals (permit number G 0129/12) and followed the “Principles of Laboratory Animal Care” (NIH publication No. 86-23, revised 1985), as well as the current version of German Law on the Protection of Animals.

**Cecal ligation and puncture surgery.** Polymicrobial sepsis was induced by cecal ligation and puncture (CLP) surgery according to a published protocol [6] and as recently reported [4]. CLP was performed on 6-8 weeks-old male C57Bl6/N mice. Mice were anesthetized with isoflurane, placed on a heating pad to assure a constant body temperature at 37°C measured by a rectal probe. After shaving and disinfection of the skin, midline laparotomy was performed, and the cecum was exposed and ligated using a non-absorbable surgical suture (Ethicon 6-0). A 21-gauge needle was used to puncture the cecum once, and a small amount of cecum content was extruded. The cecum was then replaced into the abdominal cavity, and the incisions of the peritoneum and skin were closed with two separate layers of a surgical suture (Ethicon 6-0). Sham mice were treated identically except for the ligation and puncture of the cecum. All mice were resuscitated by an *i.p.* injection of 1 ml sterile and 37°C pre-warmed Ringer’s solution. Metamizole (200 mg/kg body weight) applied twice daily *s.c.* was used as analgesic. CLP (*n* = 4-5) and sham (*n* = 4-5) mice were sacrificed 24 h, 48 h, 72 h and 96 h after surgery and *gastrocnemius/plantaris* and *tibialis anterior* were harvested for analysis. The weight of skeletal muscles were determined und tibia length was measured for reference purpose. For histology *gastrocnemius/plantaris* was dissected, mounted and frozen under cryoprotection. Muscle samples were stored at -80°C. Frozen sections with a thickness of 6 µm were cut using a Leica cryotome. **Denervation.** Adult male C57BL/6N mice (6-8 weeks of age) were used to induce skeletal muscle atrophy by dissection of the left sciatic nerve. Mice were anesthetized with isoflurane, placed on a heating pad to assure a constant body temperature at 37°C measured by a rectal probe. The sciatic nerve of the right leg was cut and a 3 mm piece was excised (denervated). The right leg remained innervated and was used as control (innervated). Mice were sacrificed at baseline, after 7 days, 14 days and 21 days of surgery, (*n* = 6 each). *Gastrocnemius/plantaris* and *tibialis anterior* were obtained, snap frozen in liquid nitrogen and stored at -80°C until further analyses. The weights of skeletal muscles were measured. Tibia length was measured and used as a reference. **Food deprivation.** Male C57BL/6N mice at the age of 6-8 weeks were food deprived for 24 h and 48 h with free access to drinking water. Mice were sacrificed after 24 h and 48 h of starvation, (*n* = 6 each). Male littermate control mice were sacrificed at 0 h (*n* = 6). *Gastrocnemius/plantaris* and *tibialis anterior* were obtained, snap frozen in liquid nitrogen and stored at -80°C until further analyses. The weights of skeletal muscles were determined. Tibia length was measured and used as a reference.

**Muscle biopsies.** For immunohistology and histological staining, muscle tissues were mounted and frozen under cryoprotection [7]. Biopsy specimens were stored at -80°C. Frozen sections with a thickness of 6 µm for immunohistochemistry and routine histology were cut using a Leica cryotome CM 3050s. For histological analyzes by Hematoxylin & Eosin staining skeletal muscle biopsy specimens were fixed using 4 % paraformaldehyde and processed.

**RNA isolation, cDNA synthesis and quantitative real-time-PCR (qRT-PCR).** Total RNA was isolated from skeletal muscle biopsies or cultured cells using TRIzol® Reagent (Invitrogen) according to the manufacturer’s protocol [7-10]. cDNA synthesis of 1 µg of RNA per sample was carried out by using the SuperScript® First-Strand Synthesis System (Invitrogen). qRT-PCR was performed using Power SYBR® Green PCR Master Mix (Applied Biosystems) and self-designed primers (for primer sequences see Additional file 1: Table S3). PCR reactions were performed in a Step-One^TM^ Plus thermocycler (Applied Biosystems) as described recently using a cDNA standard curve [1, 7, 8, 11]. To correct for potential variances between samples regarding differences in mRNA extraction and reverse transcription efficiency, gene expression was normalized to the expression of the reference gene *glyceraldehyde-3-phosphate dehydrogenase* (*GAPDH*) [1, 7, 8, 11].

**Myoblast culture and differentiation.** Cell culture experiments of murine myoblasts (C2C12 cells) were performed as described recently [12]. Differentiation of myoblasts to myotubes was induced at confluence by replacing growth medium (DME medium (1 g/l glucose) (Sigma Aldrich), 10 % fetal bovine serum (FBS) (PAA), 2 mM L-Glutamine (PAA), supplemented with Penicillin and Streptomycin (both PAA)), with differentiation medium (DMEM (1 g/l glucose), 2 % FBS, supplemented with Penicillin and Streptomycin (both PAA)). Transfection of C2C12 cells was performed using Lipofectamine® and PLUS™ reagent (both Invitrogen).

**Immunostaining of myoblasts and myotubes *in vitro*.** C2C12 myoblasts and differentiated myotubes were fixed with 4 % paraformaldehyde/PBS, permeabilized with 0.2 % Triton-X-100/PBS, and blocked with 2 % goat serum/PBS. Trim62 was detected using anti-TRIM62 (1:100, abcam), anti-DYKDDDDK tag (anti-FLAG, 1:250, Cell Signaling) or anti-myc tag (1:100, Millipore) as primary and Alexa Fluor® 488 Goat Anti-Rabbit IgG (H+L) (1:500, Invitrogen) as secondary antibody. Stained cells were embedded in ProLong Gold® Antifade Reagent that contained DAPI for nuclei stain (Invitrogen). Immunostaining was analyzed using the Leica CTR 6500 microscope and the Leica DFC 360 FX digital camera.

**Cloning of *Trim62*.** Mouse *Trim62* cDNA was amplified from an I.M.A.G.E full length cDNA clone (Source Bioscience) by PCR using primer pairs containing restriction enzyme consensus sequences: Mm_*Trim62*_XhoI forward 5’-CCG CTC GAG ATG GCG TGC AGC CTC AAG G-3’, Mm_*Trim62*_KpnI reverse 5’-GGG GTA CCG ATG CGG ACG GTG TTG ATC C-3’. The PCR product and the vector pcDNA3.1 myc-(His)_6_ (-) A (Invitrogen) were digested using respective restriction enzymes (New England Biolabs GmbH) and ligated using T4 ligase (Promega). The expression plasmid (pcDNA3.1_Mm_*Trim62*_C-myc-(His)_6_) was sequence verified and used for transfection of HEK293 cells and C2C12 cells. For cloning of the cDNA expression vector pcDNA3.1_N-FLAG_Mm_*Trim62* expressing murine Trim62 with an N-terminal FLAG tag following primers were used: Mm_*Trim62* _EcoRI_forward 5’- CGG AAT TCG CGT GCA GCC TCA AGG AC -3’, Mm_*Trim62*_XbaI_revers 5’- GCT CTA GAT TAG ATG CGG ACG GTG TTG ATC C -3’. The PCR product and the vector pcDNA3.1 (+) A (Invitrogen) containing an N-terminal FLAG tag (DYKDDDDK) were digested using respective restriction enzymes (New England Biolabs GmbH) and ligated using T4 ligase (Promega). The obtained expression vector (pcDNA3.1_Mm_*Trim62*_N-FLAG) was sequence verified and used for transfection of C2C12 cells.

**Luciferase reporter assays.** HEK293 cells were maintained in DME medium (4.5 g/l glucose) supplemented with 2 mM L-glutamine, 10 % FBS, Penicillin and Streptomycin. HEK293 cells were transfected with expression plasmids encoding Trim62 protein (pcDNA3.1_Mm_*Trim62*_C-myc-(His)_6_, 800 ng), or empty vector control (pcDNA3.1_myc-(His)_6_, 800 ng), as indicated, and the AP1 luciferase reporter (pGL3 basic containing three AP1 consensus binding sites (TGAGTCA), 100 ng), using 2.4 µg/ml Polyethylenimine (Polysciences, linear MW 2,500) for 48 h. To control for transfection efficacy pCMV lacZ (Clontech, 50 ng) was used in each sample. Luciferase activity was determined using Dual-Luciferase® Reporter Assay System (Promega) and normalized to fluorescence determined using FluoReporter® *lacZ*/Galactosidase Quantitation Kit (Invitrogen). Luciferase-to fluorescence ratios were further normalized to pGL3 basic expression plasmid.

**siRNA transfection and LPS treatment.** Differentiated C2C12 myotubes were transfected using ON-TARGET plus SMART pool Mouse 6330414G21RIK (Trim62) and ON-TARGETplus Non-targeting Control Pool (both Thermo Scientific) for 24 h. Transfections were performed according to the manufacturer’s protocol using Dharmafect 3 (Thermo Scientific). 24 h after siRNA transfection myotubes were treated with 100 µg/ml lipopolysaccharide (LPS) for 2 h. *Trim62* expression was analyzed by quantitative real-time-PCR.

**Protein extraction and immunoblotting.** Protein analyses were performed as recently published [3, 7]. Shortly, cells were lysed in ice-cold extraction buffer (1:3 wt/vol; 10 mM Tris HCl, pH 7.5, 140 mM NaCl, 1 mM EDTA, 25 % glycerol, 0.5 % sodium dodecyl sulfate (SDS), 0.5 % Nonident P-40, 0.1 mM dithiothreitol, 0.5 mM phenylmethylsulfonyl fluoride, 100 ng/ml protease inhibitor cocktail) and then cleared by centrifugation (4°C, 10 min, 12,000 x g). The supernatant was assayed for protein concentration using the Bio-Rad Protein Assay, frozen and stored at -80°C until usage. Protein (20 μg) was separated by 10 % SDS polyacrylamide gel electrophoresis (SDS-PAGE) and blotted onto PVDF membranes (Amersham Pharmacia Biotech). Membranes were incubated with specific primary antibodies: anti-Trim62 (abcam, 1:500), anti-glyceraldehyde-3-phosphate dehydrogenase (clone 6C5, Millipore, 1:30000), anti-α-tubulin (clone DM1A, 1:5000, Sigma) and secondary horseradish peroxidase (HRP) conjugated antibodies: anti-mouse IgG (Cell Signaling, 1:2000), anti-rabbit IgG (Cell Signaling, 1:2000). The anti-MuRF-1 antibody has been described earlier [3, 4, 13], and are available from www.myomedix.com. The signals were visualized with SuperSignal™ West Pico Chemiluminescent Substrate (Thermo Scientific).

**Table S1. Patients’ characteristics.**

| **Patients’ characteristics** | **All patients enrolled** | **Controls** |
| --- | --- | --- |
| **Number** | 26 | 4 |
| **Age [years]** | 54.5 (41/68) | 70 (64/75) |
| **Gender [male/female (%)]** | 19/7 (73/27) | 2/2 (50/50) |
| **BMI [kg/m^2^]** | 28 (24.1/32) | ND |
| **MRC-Score** | 3.3 (3.0/3.8) | 5.0 |
| **Diagnosis [n (%)]** | ALI/ARDS: n = 8 (30.8)  Sepsis: n = 7 (26.9)  Trauma: n = 6 (23.1)  CNS: n = 5 (19.2) | NA |
| **Survivors [n (%)]** | 21 (80.3) | 4 (100) |
| **Severity of illness**  **(at ICU admission)** | ICU scores:  SOFA: 12 (10/14)  SAPS-II: 42 (36/53) | NA |

ALI/ARDS indicates acute lung injury/acute respiratory distress syndrome; BMI, body mass index; CNS, central nervous system; ICU, intensive care unit; MRC, Medical Research Council; NA, not applicable; ND, not determined; SOFA, Sequential Organ Failure Assessment score; SAPS-II, simplified acute physiology score-II.

**Table S2. Treatment of ICU-patients during study period.**

|  | **From ICU admission to first biopsy at median day 5** | **From first to second biopsy at median day 15** |
| --- | --- | --- |
| **Number** | 26 | 14 |
| **Sedation [RASS], median** | -4.0 (-5.0/-3.0) | -2.8 (-4.0/-1.0) |
| **Hydrocortisone, sum [mg]** | 480 (0/798) | 1.4 (0.0/251) |
| **Hydrocortisone, mean per day [mg]** | 127.6 (21.5/164.2) | 1.4 (0.0/76.9) |
| **Cis-atracurium, sum [mg]** | 0.0 (0.0/10.0) | 0.0 (0.0/0.0) |
| **Norepinephrine, mean per day [mg]** | 19.8 (9.6/24.3) | 3.7 (0.8/11.0) |
| **Blood glucose between 80 and 150 mg/dl, [% of 6-hourly measured glucose levels in target range]** | 78.2 (62.5/87.5) | 86.0 (81.2/93.1) |
| **Nutrition [kcal/kg (PBW)/d]** | 15.4 (11.9/19.6) | 22.1 (19.0/26.1) |
| **Insulin [IU/d]** | 53.1 (30.8/75.6) | 50.2 (34.6/63.2) |
| **Number of patients with median ≥ 2 organ dysfunctions [n (%)]** | 14 (53.8) | 11 (50.0) |
| **Number of patients with acute renal failure [n (%)]** | 11 (42.3) | 7 (50.0) |
| **Days with septic shock (%)** | 38.8 (0.0/66.7) | 0.0 (0.0/25.0) |

RASS indicates Richmond Agitation Sedation Scale; PBW, predicted body weight. Data are shown as median (IQR) or number and percentage.

**Table S3. Primer pairs for quantitative real-time-PCR are shown.**

| **Name** | **Sequence** |
| --- | --- |
| Hs_*GAPDH* forward | 5’-AGC CAC ATC GCT CAG ACA C-3’ |
| Hs_*GAPDH* reverse | 5’-GCC CAA TAC GAC CAA ATC C-3’ |
| Hs_*TRIM62* forward | 5’-AAG TCC CTG TTC CAG GAC ATC-3’ |
| Hs_*TRIM62* reverse | 5’-GCA GTC GTC CGA CAG GAT-3’ |
| Mm_*Atrogin1* forward | 5’-AGT GAG GAC CGG CTA CTG TG-3’ |
| Mm_*Atrogin1* reverse | 5’-GAT CAA ACG CTT GCG AAT CT-3’ |
| Mm_*Gapdh* forward | 5’-ATG GTG AAG GTC GGT GTG A-3’ |
| Mm_*Gapdh* reverse | 5’-AAT CTC CAC TTT GCC ACT GC-3’ |
| Mm_*Il-6* forward | 5’-GCT ACC AAA CTG GAT ATA ATC AGG A-3’ |
| Mm_*Il-6* reverse | 5’-CCA GGT AGC TAT GGT ACT CCA GAA-3’ |
| Mm_*MuRF1* forward | 5’-CCT GCA GAG TGA CCA AGG A-3’ |
| Mm_*MuRF1* reverse | 5’-GGC GTA GAG GGT GTC AAA CT-3’ |
| Mm_*Saa1* forward | 5’-CCA GGA TGA AGC TAC TCA CCA-3’ |
| Mm_*Saa1* reverse | 5’-TAG GCT CGC CAC ATG TCC-3’ |
| Mm_*Tnfα* forward | 5’-TCT TCT CAT TCC TGC TTG TGG-3’ |
| Mm_*Tnfα* reverse | 5’-GGT CTG GGC CAT AGA ACT GA-3’ |
| Mm_*Trim62* forward | 5’-ACG ATG CCT TCG AGG AGT T-3’ |
| Mm_*Trim62* reverse | 5’-CTG TCC TGA AGG GCC TGA-3’ |

*TRIM62 indicates tripartite motif-containing 62; Saa1, serum amyloid A1; MuRF1, muscle RING-finger 1; GAPDH, glyceraldehyde-3-phosphate dehydrogenase; Il-6, Interleukin 6; Tnfa, tumor necrosis factor alpha*; Hs, *Homo sapiens*; Mm, *Mus musculus*.

**References**

1. Bierbrauer J, Koch S, Olbricht C, Hamati J, Lodka D, Schneider J, Luther-Schroder A, Kleber C, Faust K, Wiesener S *et al*: Early type II fiber atrophy in intensive care unit patients with nonexcitable muscle membrane. *Crit Care Med* 2012, 40(2):647-650.

2. Weber-Carstens S, Schneider J, Wollersheim T, Assmann A, Bierbrauer J, Marg A, Al Hasani H, Chadt A, Wenzel K, Koch S *et al*: Critical illness myopathy and GLUT4: significance of insulin and muscle contraction. *Am J Respir Crit Care Med* 2013, 187(4):387-396.

3. Wollersheim T, Woehlecke J, Krebs M, Hamati J, Lodka D, Luther-Schroeder A, Langhans C, Haas K, Radtke T, Kleber C *et al*: Dynamics of myosin degradation in intensive care unit-acquired weakness during severe critical illness. *Intensive Care Med* 2014, 40(4):528-538.

4. Langhans C, Weber-Carstens S, Schmidt F, Hamati J, Kny M, Zhu X, Wollersheim T, Koch S, Krebs M, Schulz H *et al*: Inflammation-induced acute phase response in skeletal muscle and critical illness myopathy. *PLoS One* 2014, 9(3):e92048.

5. Weber-Carstens S, Deja M, Koch S, Spranger J, Bubser F, Wernecke KD, Spies CD, Spuler S, Keh D: Risk factors in critical illness myopathy during the early course of critical illness: a prospective observational study. *Crit Care* 2010, 14(3):R119.

6. Rittirsch D, Huber-Lang MS, Flierl MA, Ward PA: Immunodesign of experimental sepsis by cecal ligation and puncture. *Nat Protoc* 2009, 4(1):31-36.

7. Fielitz J, Kim MS, Shelton JM, Latif S, Spencer JA, Glass DJ, Richardson JA, Bassel-Duby R, Olson EN: Myosin accumulation and striated muscle myopathy result from the loss of muscle RING finger 1 and 3. *J Clin Invest* 2007, 117(9):2486-2495.

8. Fielitz J, Kim MS, Shelton JM, Qi X, Hill JA, Richardson JA, Bassel-Duby R, Olson EN: Requirement of protein kinase D1 for pathological cardiac remodeling. *Proc Natl Acad Sci U S A* 2008, 105(8):3059-3063.

9. Fielitz J, van Rooij E, Spencer JA, Shelton JM, Latif S, van der Nagel R, Bezprozvannaya S, de Windt L, Richardson JA, Bassel-Duby R *et al*: Loss of muscle-specific RING-finger 3 predisposes the heart to cardiac rupture after myocardial infarction. *Proc Natl Acad Sci U S A* 2007, 104(11):4377-4382.

10. Kim MS, Fielitz J, McAnally J, Shelton JM, Lemon DD, McKinsey TA, Richardson JA, Bassel-Duby R, Olson EN: Protein kinase D1 stimulates MEF2 activity in skeletal muscle and enhances muscle performance. *Mol Cell Biol* 2008, 28(11):3600-3609.

11. Fielitz J, Hein S, Mitrovic V, Pregla R, Zurbrugg HR, Warnecke C, Schaper J, Fleck E, Regitz-Zagrosek V: Activation of the cardiac renin-angiotensin system and increased myocardial collagen expression in human aortic valve disease. *J Am Coll Cardiol* 2001, 37(5):1443-1449.

12. Mamchaoui K, Trollet C, Bigot A, Negroni E, Chaouch S, Wolff A, Kandalla PK, Marie S, Di Santo J, St Guily JL *et al*: Immortalized pathological human myoblasts: towards a universal tool for the study of neuromuscular disorders. *Skelet Muscle* 2011, 1:34.

13. Moriscot AS, Baptista IL, Bogomolovas J, Witt C, Hirner S, Granzier H, Labeit S: MuRF1 is a muscle fiber-type II associated factor and together with MuRF2 regulates type-II fiber trophicity and maintenance. *J Struct Biol* 2010, 170(2):344-353.
