# Supplementary material for: The E3 ubiquitin ligase TRIM62 and inflammation-induced skeletal muscle atrophy
Source: Crit Care. 2014 Sep 29;18(5):545. doi: 10.1186/s13054-014-0545-6 (PMC4231194; doi:10.1186/s13054-014-0545-6)
Supplement: Additional file 2: Figure S1. — Study protocol. [file 13054_2014_545_MOESM2_ESM.ppt]

## Slide 1
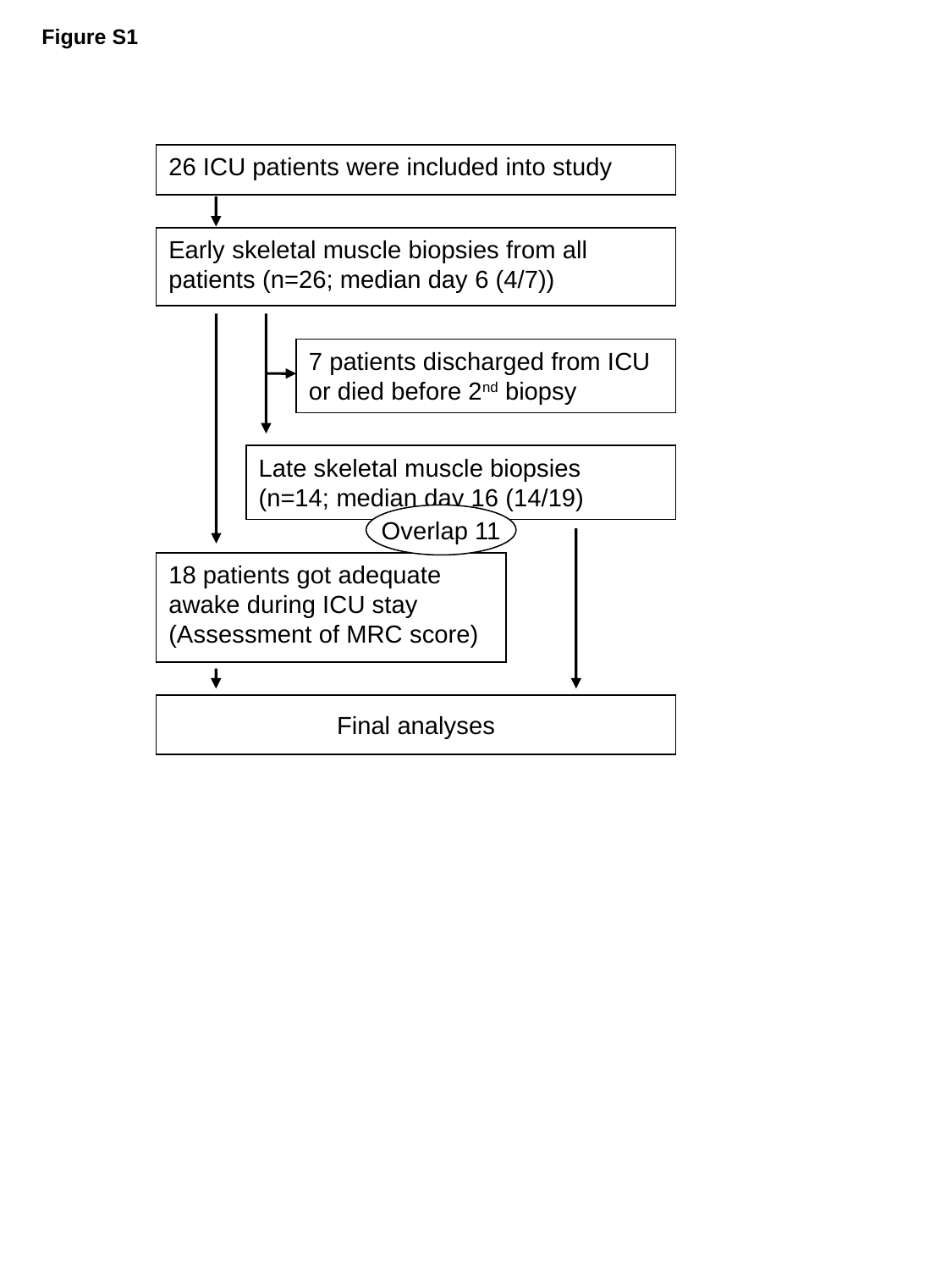

Figure S1
26 ICU patients were included into study
Early skeletal muscle biopsies from all patients (n=26; median day 6 (4/7))
7 patients discharged from ICU or died before 2nd biopsy
Late skeletal muscle biopsies
(n=14; median day 16 (14/19)
Overlap 11
18 patients got adequate
awake during ICU stay
(Assessment of MRC score)
Final analyses
