# Supplementary figures and images for: The E3 ubiquitin ligase TRIM62 and inflammation-induced skeletal muscle atrophy
Source: Crit Care. 2014 Sep 29;18(5):545. doi: 10.1186/s13054-014-0545-6 (PMC4231194; doi:10.1186/s13054-014-0545-6)

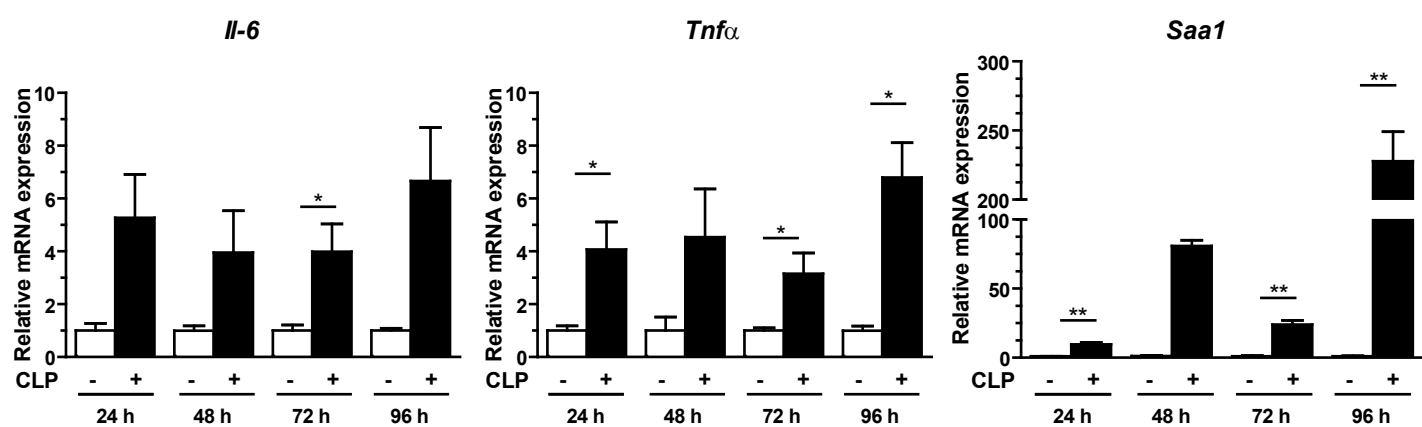

Figure S2

Supplement: Additional file 3: Figure S2. — CLP-induced inflammation and acute-phase response in liver. Quantitative RT-PCR analyses of interleukin 6 (IL-6), tumor necrosis factor α (Tnfα) and serum amyloid A1 (Saa1) expression in the liver 24 hours, 48 hours, 72 hours or 96 hours after surgery, as indicated. Glyceraldehyde 3-phosphate dehydrogenase expression was used as a reference, and data are shown as relative expression. Data presented are mean ± SEM. **P <0.01, *P <0.05. [file 13054_2014_545_MOESM3_ESM.pdf]

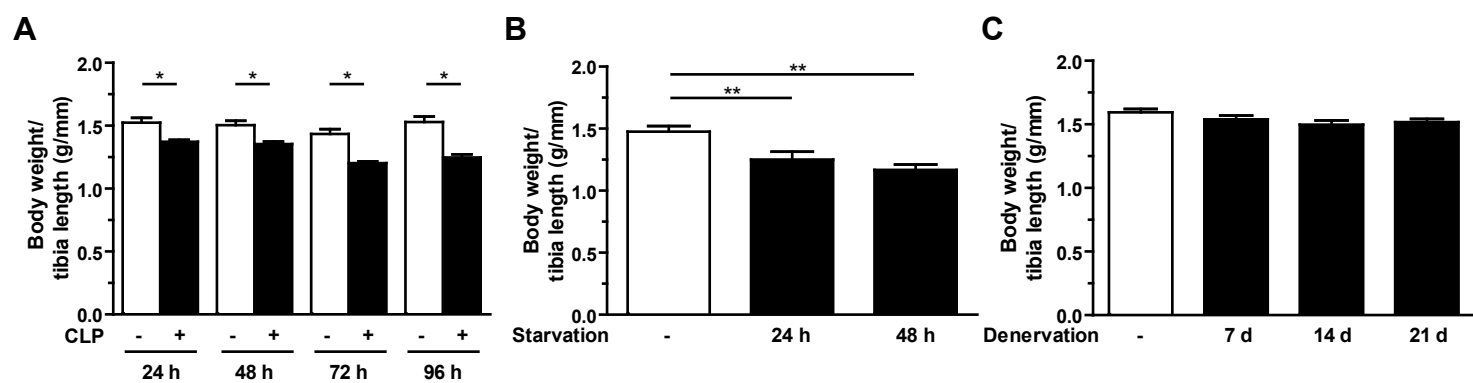

Figure S3

Supplement: Additional file 4: Figure S3. — Body weight during skeletal muscle atrophy. Body weight normalized to tibia length is shown for skeletal muscle atrophy induced by CLP (A), starvation (B) and denervation (C). Data presented are mean ± SEM. **P <0.01, *P <0.05. [file 13054_2014_545_MOESM4_ESM.pdf]
